# Reproductive Results of Selected Cat Breeds

**DOI:** 10.3390/life15071128

**Published:** 2025-07-17

**Authors:** Wojciech Wójcik, Marta Piechowska

**Affiliations:** 1 Department of Animal Breeding and Nutrition, Institute of Animal Sciences, Warsaw University of Life Sciences, 02-787 Warsaw, Poland; 2 Faculty of Animal Breeding, Bioengineering and Conservation, Warsaw University of Life Sciences, 02-787 Warsaw, Poland; s214299@sggw.edu.pl

**Keywords:** feline, queen, reproductive results, kittens, multiparous queens

## Abstract

The main goal of purebred cat breeding is to produce healthy offspring while maintaining breed purity. Pedigree cat breeders are affiliated with international federations that enforce similar breeding regulations, which helps prevent the overexploitation of cats in breeding. The minimum age for allowing a cat to breed is at least 10 months. This study aimed to analyze the breeding performance of three cat breeds: Maine Coon (MCO), British Shorthair (BSH), and Devon Rex (DRX). These breeds are classified as late-maturing (MCO), medium-late maturing (BSH), and early-maturing (DRX). The analysis was based on pedigree data obtained from the Polish Felinological Association, which operates under the auspices of the World Cat Federation. In total, data from 1016 litters (453 MCO, 453 BSH, and 110 DRX) were analyzed. Breeds differed significantly in age at first birth (*p* = 0.041), mean interval between litters (*p* < 0.01), and mean number of kittens per litter (*p* < 0.01). Breed effects were also noted for the mean interval between births (*p* < 0.01), mean number of kittens per litter (*p* < 0.01), and the total number of kittens sired by the mother (*p* = 0.007). Within each breed, differences were found in the sex ratio of litters, with a predominance of males in DRX (*p* = 0.049), MCO (*p* = 0.003), and overall breeds (*p* = 0.036). In contrast, the BSH breed showed no significant difference in the sex ratio of litters (*p* = 0.455). In both the MCO and DRX breeds, the lowest average interval between litters was observed in females that gave birth to their first litter early compared to those that gave birth later. The total number of kittens raised by the mother was highest in the MCO breed (*p* ≤ 0.05), while the shortest litter spacing was found in the DRX breed (*p* ≤ 0.05).

## 1. Introduction

The main goal of pedigree cat breeding is to enhance specific traits through proper mating of individuals and to produce healthy offspring [1,2,3]. Pedigree cats are maintained with breed purity and are registered with felinological federations, which keep pedigree books for each breed. An essential element of proper breeding documentation is the registration of kittens, which helps control restrictions on the use of cats in breeding and monitors the frequency of births over time. Female cats are typically allowed to breed once they reach 10 months of age (sexual maturity). This is in accordance with the breeding regulations of the Polish Felinological Association Part IV Cats and Kittens in Breeding & 27 [4]. It is recommended that she give birth three times within 2 years, although four births within 2 years is also permissible [5,6]. Cats can be distinguished by the age at which they reach sexual maturity: some breeds mature later than 10 months, others reach puberty moderately later, and some mature much earlier. Larger cats tend to reach maturity later, usually occurring when they reach 80% of their target body weight. Large cats such as Maine Coons (MCOs) not only have a longer puberty but also have different reference ranges as to the course of delivery and hematological and biochemical blood reference values. Late-maturing breeds include MCOs—about 10–16 months old, while medium-maturing breeds include British Shorthairs (BSH)—puberty occurs around 9–12 months old. MCOs, which originated in the harsh climate of Maine in the United States, and BSHs, which originated in the British Isles, are valued for their calm temperament and hunting abilities [2,7,8,9,10,11,12,13]. In contrast, early-maturing breeds include Siamese and Oriental cats, as well as some breeds such as Bengal and Devon Rex (DRX)—about 6 months old. The DRX, a young breed resulting from the fixation of a single point mutation affecting hair structure [6,13], has gained increasing popularity. In many catteries, DRX cats are the second most commonly bred breed. In the Fédération Internationale Féline d’Europe (FIFe), the number of registered DRX cats rose from 3437 in 2019 (ranked eighth in FIFe breed popularity) to 4438 in 2023 (ranked sixth), marking an increase of 0.9% [14,15]. In comparison, the MCO was ranked No. 1 during this period, with 25,459 cats registered in 2019 and 24,691 in 2023, reflecting a 1.5% decrease. The BSH, ranked second in both 2019 and 2023, saw a slight increase in the number of registered cats, rising from 16,071 in 2019 to 17,629 in 2023 [14,15].

Based on breeding regulations, it was hypothesized that MCO cats, classified as late-maturing, and BSH cats, classified as moderately late-maturing, should not be mated too early (before the age of 10 months). On the other hand, DRX cats, classified as early-maturing, should not be mated too late (after 18 months of age). Representatives of these breeds differ significantly in exterior characteristics and body weight. MCO cats are large, BSH cats are medium-sized, and DRX cats are relatively small. Another important consideration is the average size of the female. Breeders believe that heavier females tend to have larger abdominal volumes. This, in turn, provides more space for the pregnant uterus, which may increase the chances of delivering a healthy and numerous litter. Insufficient space in the uterus, however, can restrict the ability of the fetuses to develop properly, a condition known as intrauterine growth restriction (IUGR). The IUGR effect is common in twin pregnancies in humans and in multifetal pregnancies in animals, including cats [16,17].

It was also hypothesized that large cats with a longer development and maturation period (MCO and BSH) give birth to larger litters than cats that mature early but are much finer (DRX). The aim of this study was to analyze data obtained from pedigrees issued by the Polish Felinological Federation, which operates under the auspices of the World Cat Federation (WCF), regarding the reproduction of three domestic cat breeds (DRX, BSH, and MCO). According to the breeding regulations, four births within 2 years are allowed, but three births during this period are recommended.

## 2. Materials and Methods

The material for the study consisted of data obtained from the herd book maintained by the Polish Felinological Federation, which operates under the auspices of the WCF. The pedigree data for BSH, MCO, and DRX cats were sourced from randomly selected catteries between 2006 and the end of 2023. A total of 453 litters for BSH cats and 453 litters for MCO cats were analyzed. For DRX cats, a breed that has only recently gained popularity, pedigree data were collected from 110 litters. The data included the total reproductive results of 327 breeding cats: 158 BSH cats, 136 MCO cats, and 33 DRX cats.

The following information was available in the litter registration data:

- Number of registered offspring (alive at the time of litter registration, approximately 7 to 8 weeks of age). This analysis does not include data on early mortality, which can range from a few to several percent.
- Gender of the registered offspring.
- Date of birth of the litter.
- Data of the mother (name with breeding nickname and date of birth).
- Data of the father (name with breeding nickname).
- Color of the parents and offspring.

### 2.1. Age of the Female on the Day of Each Birth and the Interval Between Births

Based on the mother’s birth date and the birth date of the litter, the mother’s age on the day of the first birth was calculated. Then, for each subsequent litter, the mother’s age on the day of each litter, expressed in days, was determined. Using the mother’s age on the day of each subsequent birth, the interval between each litter was calculated. For each breed, the average number of litters born to cats of that breed, the average interval between births, and the sex distribution for each breed were calculated for all litters.

### 2.2. Date of First Birth

Based on pedigree data (the female’s age and the date of her first birth), the approximate date of mating was determined. It was established that the minimum length of a normal pregnancy is 60 days, and this value was subtracted from the female’s age on the date of her first birth. This allowed for the division of all females into four groups:

- Early first birth (EFB): Female cats that were successfully mated before 300 days of age (i.e., before 10 months old) and gave birth before 360 days of age—mating below the minimum age (10 months old).
- Optimal first birth (OFB): Females that were successfully mated between 301 and 420 days of age (i.e., between 10 and 18 months old) and gave birth between 361 and 480 days of age—recommended age for mating.
- Late birth (LFB): Females that were successfully mated between 421 and 660 days of age (i.e., between 18 and 24 months old) and gave birth between 481 and 720 days of age—late age for mating.
- Very late first birth (VLFB): Females that were successfully mated after 660 days of age (i.e., after 24 months old) and gave birth after 720 days of age—very late age for mating.

The mean number of kittens and the sex distribution of kittens in litters were calculated for each of these subsets. Then, the average interval between consecutive litters was calculated for females based on the date of successful mating. In addition, the sex distribution of all litters and the total number of live kittens from females during the analyzed period (ranging from 2 to 11 litters born per female) were determined.

### 2.3. Interval Between Litters

Depending on the length of time between births, the intervals were divided into three groups:

- Short time (STe): An interval shorter than the regulatory period, i.e., less than 180 days between two litters.
- Regular time (RTe): The regulatory interval, i.e., between 181 and 360 days between two litters.
- Long time (LTe): An interval longer than the regulatory period, i.e., more than 361 days between two litters.

Based on the length of birth intervals, their effect on litter size and the sex distribution of kittens was determined.

### 2.4. Total Number of Kittens from the Mother

Among all the female cats, those that gave birth to five or more litters were selected and referred to as “feline record-breakers.” Based on the number of births and the number of kittens, the total number of kittens per female cat, the average number of kittens per litter, the interval between births, and the sex distribution among the offspring were determined.

### 2.5. Statistical Methods

The results are presented as means and standard deviations (SD). To evaluate the influence of the various experimental factors, univariate or bivariate analyses of variance were performed, with results presented as *p*-values for individual factors and their interactions.

For multiple comparisons between breeds or between time intervals, one-way ANOVA was applied based on the following models:

$$Y_{ij}=\mu +A_{j}+e_{ij\;}or\;Y_{jk}=\mu +B_{k}+e_{ik}$$

A two-way ANOVA was applied to evaluate the effects of breed and time interval, as well as their interaction, according to the following model:

$$Y_{ijk}=\mu +A_{j}\;or\;B_{k}+{\left[AB\right]}_{jk}+e_{ijk}$$

where *Y* is the dependent variable, *μ* is the general mean, *A_i_* represents the effect of the breed, and *B_k_* represents the effect of the time interval. The results of the two-way ANOVA are presented as *p*-values.

In addition, the pooled standard error of the mean (SEM) was presented as a parameter of variation. Multiple comparisons of means were made using Duncan’s multiple range test. To evaluate the relationships between selected traits (such as the age of the cat on the day of birth, the interval between litters, and the average number of kittens in these litters), Pearson correlation analysis was performed. The analyses were performed using Statistica 13.3 (TIBCO). A significance level of 0.05 was assumed for all analyses [18].

## 3. Results

The characteristics of the raw data are presented in Table 1. The raw data is available as Supplementary Materials. The following information is provided for each of the three breeds:

- Number of females giving birth to each subsequent litter.
- Average age of the cat at each subsequent birth.
- Average number of kittens in each successive litter.
- Ratio of male and female kittens born in each successive litter.
- Average interval between litters. The longest duration recorded was for one MCO cat, which gave birth to a total of 11 litters, with the last litter born at 2645 days (7 years, 3 months). However, the DRX cat gave birth to 10 litters over 3219 days (8 years, 10 months).

**Table 1:** Characteristics of raw data.

Litter	Breed	Number of Queens	Average Age of Queen (Days)	Average Number of Kittens in Litter *	Ratio of Females in a Litter (%)	Ratio of Males in a Litter (%)	Average Time Between Successive Litters (Days) **	
1	BSH	151	514	4.2	48.7	51.3		
	MCO	131	516	4.2	43.2	56.8		
	DRX	33	423	3.4	41.4	58.6		
2	BSH	109	860	4.3	46.7	53.3	1/2	355
	MCO	100	787	4.7	50.9	49.1		283
	DRX	26	717	3.0	45.5	54.5		261
3	BSH	73	1238	4.2	50.0	50.0	2/3	351
	MCO	70	1079	4.9	45.1	54.9		298
	DRX	19	1045	2.9	56.8	43.2		272
4	BSH	57	1643	4.0	49.7	50.3	3/4	404
	MCO	54	1374	4.7	44.1	55.9		302
	DRX	14	1303	3.1	34.7	65.3		271
5	BSH	28	1877	3.8	49.4	50.6	4/5	292
	MCO	37	1646	5.3	44.7	55.3		316
	DRX	7	1395	3.4	60.2	39.8		215
6	BSH	16	2206	4.1	57.0	43.0	5/6	384
	MCO	29	1944	4.9	46.0	54.0		317
	DRX	4	1910	3.8	43.8	56.3		467
7	BSH	8	2485	4.5	42.4	57.6	6/7	351
	MCO	16	2131	5.1	56.8	43.2		263
	DRX	4	2177	2.8	22.5	77.5		267
8	BSH	3	2539	3.3	78.3	21.7	7/8	277
	MCO	8	2158	5.0	48.8	51.2		172
	DRX	1	2542	2.0	0.0	100		269
9	BSH	2	3121	3.0	70.0	30.0	8/9	362
	MCO	3	2474	3.7	36.5	63.5		262
	DRX	1	2757	3.0	33.3	66.7		215
10	BSH	-	-	-	-	-	9/10	-
	MCO	2	2263	5.5	45.0	55.0		216
	DRX	1	3219	1.0	0.0	100		462
11	BSH	-	-	-	-	-	10/11	-
	MCO	1	2645	3.0	33.3	66.7		175
	DRX	-	-	-	-	-		-

*—The average number of kittens reared by the mother in each successive litter. **—is the average time between consecutive litters (days), i.e., 1 and 2, 2 and 3, etc.

### 3.1. Effects of Breeds

**Figure 1:**
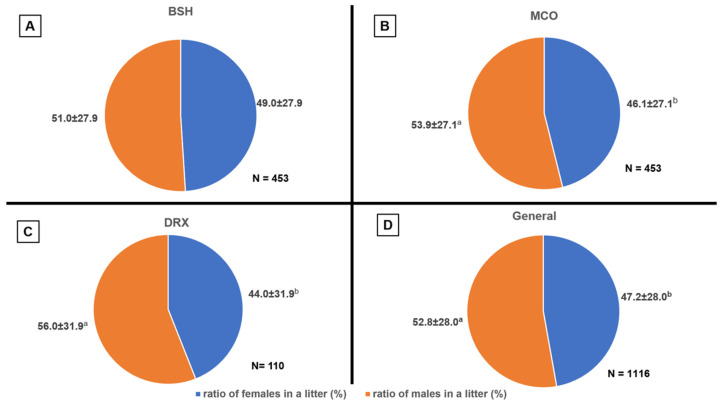
Proportion of females to males in each successive litter in three breeds ((**A**) British shorthair, (**B**) Maine coon, (**C**) Devon rex and (**D**) general results) (litters 2–11) (average ± SD). Different letters (a, b) indicate statistically significant differences between averages for differences in breed: for BSH (*p*-values = 0.455), MCO (*p*-values = 0.003), DRX (*p*-values = 0.049), and for general results (*p*-values = 0.036).

The effect of breed on the age of the first litter was confirmed (*p* = 0.041). DRX cats gave birth to their first litter the earliest, at approximately 423 days of age. BSH and MCO cats gave birth to their first litter 90 and 86 days later, respectively (Table 2). Breed had a significant effect on the length of time between litters (*p* < 0.001), the average number of kittens per litter (*p* < 0.001), and the total number of kittens for the entire lifetime (*p* = 0.007) (Table 2 and Table 3). However, breed did not significantly affect the average number of kittens in the first litter (*p* = 0.070) (Table 4). The interval between litters was generally longest for BSH females, with the exception of the VLFB group, where the length was comparable across all breeds (*p* > 0.05). BSH and MCO females in the LFB group also had similar intervals between litters, which were longer than those for DRX cats (Table 2 and Table 3). The highest number of kittens reared per litter, 5.3, was observed in MCO EFB cats (Table 3), as well as in MCO cats with a short interval between litters (5.29) (Table 5). The fewest kittens reared per litter were observed in DRX cats (Table 3 and Table 5) and in BSH cats with short intervals between litters (Table 5). Consequently, the total number of individual litters was highest in the MCO breed and lowest in the DRX breed (Table 2). The total number of kittens reared over a female’s lifetime was highest for MCO EFB cats, while DRX EFB cats had the fewest kittens, with BSH EFB cats producing comparatively few as well (Table 3). The effect of breed on the proportion of males and females in litters was not confirmed in general (both: Table 4; *p* = 0.238, Table 5; *p* = 0.541, Table 6; *p* = 0.186). However, an additional analysis of the sex structure in litters across all breeds showed that, in general, the proportion of males was higher than that of females (*p* = 0.036). The largest difference was observed in the MCO breed (*p* = 0.003), with a less pronounced but still significant predominance of males in the DRX breed (*p* = 0.049). In the BSH breed, the proportion of males and females was similar (*p* = 0.455) (Figure 1). Reproductive efficiency in females that gave birth to five or more litters is shown in Table 6. The correlations found earlier were also confirmed for multiparous females. Breed had a significant effect on all analyzed traits (*p* < 0.001), except for the proportion of males and females per litter (*p* = 0.186). The highest number of kittens per litter (5.2) and the highest total number of kittens reared (33.6) were observed in MCO females that gave birth five or more times. DRX cats had the fewest kittens per litter, but the total number of kittens reared in both DRX and BSH breeds was similar (*p* > 0.05). The longest interval between litters was confirmed for the BSH breed, while cats giving birth to five or more litters in both the MCO and DRX breeds had similar birth frequencies (Table 6).

### 3.2. Effects of First Birth

The age of the first birth had no effect on any of the analyzed traits when considering all cats (Table 4), nor when analyzing cats that had only one litter during the study period (Table 3). However, when analyzing the effect of the first birth while excluding cats that had only one litter, the lowest average interval between litters was observed in the MCO and DRX breeds for EFB cats, compared to those giving birth to their first litter later (OFB, LFB, and VLFB). In the MCO breed, the average number of kittens per litter decreased with the age of the first birth, but a significant difference was confirmed only between EFB and VLFB cats. The highest total number of kittens was observed in MCO EFB cats compared to the other groups within the breed.

### 3.3. Effects of Time Between Following Litters

Litter interval length had no significant effect on either the average number of kittens per litter (*p* = 0.139) or the sex ratio (*p* = 0.225) (Table 5). However, a higher number of kittens per litter was observed in the MCO breed in cats with a short interval between births compared to those with a long interval (Table 5).

### 3.4. Effects of Interaction

No effect of the breed × queen age interaction was observed for the average number of kittens per litter, both for all females (*p* = 0.954) and when excluding females that produced only one litter during the study period (*p* = 0.757) (Table 3 and Table 4). Similarly, the mean interval between consecutive litters, the total number of kittens from the mother, and the proportion of males and females per litter were not influenced by the breed × queen age interaction (*p* > 0.05) (Table 3 and Table 4).

The breed × time between litters interaction was also not significant for the average number of kittens per litter (*p* = 0.406) or the proportion of males and females per litter (*p* = 0.956) (Table 5).

### 3.5. Effects of Correlations

In all cat breeds, a positive and significant correlation between the queen’s age and the interval between litters was confirmed (*p* ≤ 0.05). As the age of the cat increased, the time between litters also increased (Table 7). It was also confirmed that longer intervals between births were associated with fewer kittens per litter, as evidenced by significant negative correlations in the BSH (r = −0.125) and MCO (r = −0.130) breeds (*p* < 0.05). In DRX cats, the correlation between the interval between litters and the number of kittens per litter was also negative (r = −0.179), but its statistical significance was not confirmed (*p* > 0.05). In all breeds, there was a trend toward fewer kittens per litter in older cats (negative correlations), but these trends were not significant (*p* > 0.05) (Table 7).

## 4. Discussion

The most important result of the study is that MCO cats, despite being considered a late-maturing breed, rear more litters and have shorter intervals between litters when mated early. It is likely that the classification of MCO as a late-maturing breed, especially due to their prolonged somatic development, influences the belief that they should be mated later. However, the pedigree data analyzed suggest that MCO females who mated earlier achieve better results in terms of the number of reared kittens and shorter litter intervals. This may be because more numerous litters of kittens occupy almost all the available nipples, but the mother’s milk production quickly proves insufficient, which may lead to the mother’s rapid weight loss and lower milk production and stimulate the young kittens to begin consuming the available solid food. In a study by Ludwiczak et al. [19], it was shown that a female rabbit has a higher daily milk production when rearing 10 than when rearing 8 young [19]. With prolonged lactation or large litter sizes, the milk produced by the female becomes less valuable (lower protein and fat content) due to the burden of high milk production, resulting in a decrease in condition [20]. If the female is in poorer condition at the time of mating (less body fat), better results can be observed regarding ease of conception, number of fetuses, and ease of delivery compared to females in better condition and with more fat at mating. This has been shown in many species with multifetal pregnancies (pigs, dogs, or rabbits) [2,21,22,23,24]. The similar number of kittens reared by BSH and DRX cats is due to the compensation between the number of kittens reared per litter and the time between births. DRX cats, despite having smaller litters, gave birth with shorter intervals than BSH cats. In the results obtained for DRX cats, the earlier age of first birth may be attributed to the temperament of DRX cats and the speed of maturation of the breeds from which DRX cats are descended, namely the local cats in the UK and the Sphynx. DRX females are small in stature, which, combined with limited abdominal space, limits the possibility of very large litters compared to larger breeds such as BSH or MCO cats, which are the largest domestic cat breeds [7,16,17]. Devon Rex cats had a shortened period between pregnancies, averaging just over 9 months, in accordance with the restrictions of the felinological federations. According to the observations of Ng et al. [5], females in catteries gave birth to an average of 2.3 litters per year. The increased number of births was in response to the needs of cattery buyers, but also violated breeding regulations [5]. Longer intervals between births, exceeding 12 months, have been observed in BSH cats. This may be due to the tendency of BSH females to become overweight, leading to reproductive problems. Unfortunately, data for queen weight was not available for inclusion as a covariate in the present study. On average, neutered females weigh 0.2 kg more than breeding females (castrated 5.8 kg vs. 5.6 kg uncastrated females). The problem of obesity affects many cats, including those kept ex situ [25,26]. In MCO females, despite the long maturation period, long intervals between litters are not observed, along with relatively larger litters compared to other domestic cat breeds [27,28]. The results obtained regarding the number of litters are consistent with those presented by Fournier et al. [27], confirming the number of litters in the population of these breeds.

In the observed results, there was no effect of breed on the proportion of females and males in litters. However, within breeds, differences were noted in the proportion of males to females, with a predominance of males. The results (Figure 1) regarding sex distribution are consistent with those obtained by Mugnier et al. [17], where 3547 kittens born in 932 litters were analyzed, showing a ratio of 1894 males to 1653 females, or 53.4% males and 46.6% females. Similar results in the MCO breed were obtained by Socha et al. [28], where the ratio of males to females was 59% to 41%. In addition, the sex ratio in the results obtained does not align with the Williams model, which suggests that a female who gives birth to fewer litters will have a preponderance of females in them, while more numerous litters are expected to have a preponderance of males. This may be due to evolutionary adaptations of the domestic cat (*Felis catus*) to lead a solitary lifestyle, where males have a greater opportunity for reproductive success, impregnating multiple females across a large territory, as confirmed by the Trivers-Willard Model [29,30]. The Trivers-Willard Model suggests that in polygynous species, where males show greater variation in individual fitness, mothers in good condition and favorable environmental conditions should invest more in sons, provided that sons benefit more than daughters. This occurs when offspring quality correlates with adult quality, and offspring quality is a good indicator of maternal quality [30,31]. The question arises whether the unequal sex ratio is due to the Trivers-Willard model or if it results from lethal sex-linked genes, which are more common in inbred populations and disrupt the sex ratio. In Felis catus, this may be linked to the presence of recessive genetic defects on the X chromosome that cause lethal diseases (early embryo death and resorption). Differences in sex ratios between litters of different breeds may also result from inbreeding within these breeds. After the period of military operations in the 20th century, efforts were made to recreate MCO and BSH populations from a limited number of individuals, while DRX cats are descended from a single individual who developed a mutation affecting hair structure [7,32,33].

Late births in female cats over 720 days of age may result from reproductive problems in purebred animals, as highlighted by many authors [5,28,33,34]. A trend of fewer litters is noticeable in cats giving birth to their first litter at a later age. The analysis refers to the number of reared kittens (for which pedigrees were issued), not accounting for early kitten mortality. Future studies can analyze the timing of births in cats mated at different ages, as well as the effect of a cat’s age on the mother’s care of her offspring and their survival rates.

Cats with significantly longer intervals between births tend to rear fewer kittens. This may result from the extended interval allowing the cat to rebuild her condition and accumulate fat reserves, which, if excessive, can adversely affect reproductive function [25,33]. Both excessive and insufficient adipose tissue levels can negatively impact reproductive performance. In mice, the detrimental effects of both accumulated adipose tissue and its deficiency on proper reproductive system functioning have been demonstrated [35].

So far, reproductive longevity in purebred domestic cats has not been studied. Longevity may be influenced by factors such as ovulation frequency and maternal mating [34,36]. Among cats giving birth to more than five litters, a shorter interval between litters can be observed in all breeds. It is possible that easy births and the lack of pregnancy issues were factors in keeping cats in breeding for longer periods, thus shortening the interval between litters. Based on the correlations shown (Table 7), it can be concluded that the older the cat, the longer the interval between births tends to be. In addition, the number of kittens born, and thus reared, decreases with age, while the time between births increases. These factors likely influence the decision to withdraw a particular female cat from the breeding program, significantly reducing the number of cats giving birth to their fifth and subsequent litters. As reported by Sperkes et al. [37], there is no clear evidence of a decline in fertility in older female domestic cats. However, by analyzing the results of the present study (Table 1), it can be concluded that from the seventh litter onward, there is a downward trend in the number of kittens born. As the authors noted, the number of mothers kept for such a long time is too small to provide a basis for statistical analysis [37]. This finding is consistent with our own results. In the future, it would be necessary to analyze only those cats that gave birth to at least seven litters, and with a larger dataset, more reliable conclusions can be drrawn, allowing for results that are more representative of the population.

An important element of working on datasets is the lack of verification of their reliability. Among any group of people, even among breeders registered with world-renowned felinological associations, there may be breeders who will register combined litters (two litters registered per female) or, in the case of sickly or weaker litters, will deliberately delay the registration of kittens (fear of kitten mortality). Therefore, with historical data, it is important to work on a large number of data to negate the effect of fraudulent practices.

## 5. Conclusions

The results presented correlations on the reproductive performance of cats from the three breeds. So far, before this work, authors have not analyzed reproductive correlations in domestic cats.

Based on the results (grouped as EB, Ste, and RTe), it can be concluded that MCO and BSH cats should be mated early, even if they experience frequent estrus before or just after 10 months of age. On the other hand, cats from all the analyzed breeds (MCO, BSH, and DRX) should not be mated too late. Only among BSH cats was there a trend of more reared kittens in the VLB group. Perhaps this is due to the care of older cats. In the future, data from the day of birth should be taken into account, as well as consideration of early kitten mortality and postnatal losses.

The obtained results not only characterize selected parameters of reproduction in purebred cats but also provide interesting insights for both breeders of these breeds and representatives of felinological federations. The reproductive characteristics of purebred cats are a dynamic aspect of companion animal breeding and can help to illustrate the directions in which modern felinology is evolving.

## Figures and Tables

**Table 2:** Comparison of the reproductive performance of feline queens of three breeds (average ± SD).

Breed	Number of Queens	Average Age of Cat at First Litter	Average Time Between Successive Litters	Average Number of Kittens in Litter
BSH	158	^a^ 513.5 ± 153.2	^b^ 358.7 ± 148.5	^b^ 4.15 ± 1.77
MCO	136	^a^ 509.1 ± 194.0	^a^ 291.4 ± 131.9	^c^ 4.66 ± 2.19
DRX	33	^b^ 423.3 ± 117.9	^a^ 275.2 ± 134.8	^a^ 3.13 ± 1.26
General	327	503.2 ± 170.4	318.0 ± 143.7	4.27 ± 1.97
*p*-values *		0.041	<0.001	<0.001

a, b, c—values in columns marked with different letters are significantly different at *p* ≤ 0.05. Number of queens—all females from which litters were registered in the analyzed period; *—*p*-value for main effect of the factor (breed) based on ANOVA.

**Table 3:** Effect of a queen’s age at first litter on the average interval between successive litters, the average number of kittens per litter, and the total (lifetime) number of kittens per queen over the analyzed period (litters 2–11) (average ± SD).

Breed	Age of Queen at First Litter	Number of Litters	Average Time Between Litters	Average Number of Kittens in Litter	Total Number of Kittens
BSH	EB	45	^Y^ 350.1 ± 154.7	^Y^ 4.40 ± 1.74	^X^ 14.14 ± 5.55
	OB	199	^Y^ 356.3 ± 144.7	^Y^ 4.26 ± 1.86	15.98 ± 10.36
	LB	91	^Y^ 373.5 ± 159.6	^Y^ 3.91 ± 1.55	12.71 ± 5.36
	VLB	39	^X^ 346.2 ± 168.2	^Y^ 4.05 ± 1.72	17.56 ± 8.00
MCO	EB	66	^Xa^ 257.8 ± 117.4	^Zb^ 5.30 ± 2.13	^Yb^ 26.92 ± 14.28
	OB	244	^Xb^ 300.6 ± 125.5	^Yab^ 4.72 ± 2.15	^a^ 18.87 ± 12.43
	LB	55	^XYb^ 307.1 ± 131.3	^Yab^ 4.67 ± 2.33	^a^ 17.13 ± 10.58
	VLB	37	^Xb^ 305.9 ± 183.1	^Ya^ 4.27 ± 2.38	^a^ 15.80 ± 13.01
DRX	EB	20	^Xa^ 239.3 ± 104.5	^X^ 3.10 ± 1.37	^X^ 10.33 ± 5.75
	OB	44	^Xb^ 279.8 ± 132.2	^X^ 3.32 ± 1.25	13.27 ± 6.56
	LB	22	^Xb^ 293.5 ± 142.9	^X^ 3.05 ± 1.21	13.40 ± 7.02
	VLB	19	^Xb^ 277.8 ± 161.5	^X^ 2.89 ± 1.33	13.75 ± 11.76
Pooled SEM			140.7	1.93	10.36
Main effect		*p*-values			
	Breed *		<0.001	<0.001	0.007
	Queen’s age **		0.278	0.203	0.793
	Breed x Queen’s age ***		0.959	0.757	0.207

Age of queen at first litter: EB—Early birth: Queens that were successfully mated before the age of 10 months (i.e., before 300 days of age) and gave birth before 360 days of age; OB—Optimal birth: Queens that were successfully mated between 10 and 16 months of age (i.e., between 301 and 480 days of age). These queens gave birth between 361 and 480 days of age; LB—Late birth: Queens that were successfully mated between 18 and 24 months of age (i.e., between 481 and 720 days of age). These queens gave birth between 481 and 720 days of age; VLB—Very late birth: Queens that were successfully mated after 22 months of age (i.e., beyond 720 days). These queens gave birth after 721 days of age. Different letters (a, b) indicate statistically significant differences between averages for different age groups within the same breed, while different letters (X, Y, and Z) indicate statistically significant differences between breeds within the same age group; * *p*-value for main effect of the first factor (breed) based on ANOVA; ** *p*-value for main effect of the second factor (queen’s age) based on ANOVA; *** *p*-value for interaction of two factors based on ANOVA.

**Table 4:** Effect of breed and age of the queen on the number of kittens and the ratio of females to males in the first litter (average ± SD).

Breed	Age of Queen at First Litter	Number of Queens	Average Number of Kittens in Litter	Ratio of Females in a Litter (%)	Ratio of Males in a Litter (%)
BSH	EFB	19	4.68 ± 1.60	41.8 ± 26.3	58.2 ± 26.3
	OFB	79	4.27 ± 2.00	50.2 ± 27.3	49.8 ± 27.3
	LFB	35	3.89 ± 1.62	47.2 ± 32.4	52.8 ± 32.4
	VLFB	18	4.00 ± 1.41	52.3 ± 34.4	47.7 ± 34.4
MCO	EFB	17	4.65 ± 1.87	40.4 ± 25.3	59.6 ± 25.3
	OFB	78	4.35 ± 2.09	44.0 ± 29.3	56.0 ± 29.3
	LFB	20	3.60 ± 1.60	46.3 ± 28.6	53.7 ± 28.6
	VLFB	15	3.47 ± 1.81	37.3 ± 28.9	62.7 ± 28.9
DRX	EFB	10	3.40 ± 1.51	43.0 ± 34.9	57.0 ± 34.9
	OFB	13	3.62 ± 1.04	43.2 ± 23.9	56.8 ± 23.9
	LFB	6	3.17 ± 0.98	44.4 ± 35.6	55.6 ± 35.6
	VLFB	4	3.00 ± 0.82	27.1 ± 20.8	72.9 ± 20.8
Pooled SEM			1.83	29.1	29.1
Main effect		*p*-values			
	Breed *		0.070	0.238	0.238
	Queen age **		0.191	0.679	0.679
	Breed x Queen age ***		0.954	0.857	0.857

Age of queens at first litter: EFB—Early first birth: Queens that were successfully mated before the age of 10 months (i.e., before 300 days of age) and gave birth before 360 days of age; OFB—Optimal first birth: Females that were successfully mated between 301 and 420 days of age (i.e., between 10 and 18 months of age). These cats gave birth between 361 and 480 days of age; LFB—Late first birth: Females that were successfully mated between 421 and 660 days of age (i.e., between 18 and 24 months of age). These cats gave birth between 481 and 720 days of age; VLFB—Very late first birth: Females that were successfully mated after 660 days of age (i.e., over 24 months of age). These cats gave birth after reaching 720 days of age; * *p*-value for main effect of the first factor (breed) based on ANOVA; ** *p*-value for main effect of the second factor (queen’s age) based on ANOVA; *** *p*-value for interaction of two factors based on ANOVA.

**Table 5:** Effect of breed and interval between litters on the number of kittens and the proportion of females to males in each successive litter (litters 2–11) (average ± SD).

Breed	Time Between Litters	Average Number of Kittens in Litter	Ratio of Females in a Litter (%)	Ratio of Males in a Litter (%)
BSH	STe	^X^ 3.84 ± 1.86	53.4 ± 31.9	46.6 ± 31.9
	RTe	^Y^ 4.27 ± 1.55	48.4 ± 25.2	51.6 ± 25.2
	LTe	^Y^ 3.91 ± 1.98	50.6 ± 30.5	49.4 ± 30.5
MCO	STe	^Yb^ 5.29 ± 2.22	48.3 ± 22.6	51.7 ± 22.6
	RTe	^Zab^ 4.83 ± 2.06	45.4 ± 26.2	54.6 ± 26.2
	LTe	^Ya^ 4.56 ± 2.52	49.7 ± 30.4	50.3 ± 30.4
DRX	STe	^X^ 2.80 ± 1.37	50.3 ± 33.7	49.7 ± 33.7
	RTe	^X^ 3.25 ± 1.21	42.3 ± 32.6	57.7 ± 32.6
	LTe	^X^ 2.56 ± 1.36	48.4 ± 36.0	51.6 ± 36.0
Pooled SEM		1.94	28.0	28.0
Main effect		*p*-values		
	Breed *	<0.001	0.541	0.541
	Time between litters **	0.139	0.225	0.225
	Breed × time between litters ***	0.406	0.956	0.956

Time between litters: STe—Short time: An interval shorter than the regulatory time, i.e., less than 180 days between two litters; RTe—Regular time: A regulatory interval between 181 and 360 days between two litters; LTe—Long time: An interval longer than the regulatory time, i.e., more than 361 days between two litters. Different letters (a, b) indicate statistically significant differences between averages for different age groups within the same breed, while different letters (X, Y, and Z) indicate statistically significant differences between breeds within the same age group; * *p*-value for main effect of the first factor (breed) based on ANOVA; ** *p*-value for main effect of the second factor (time) based on ANOVA; *** *p*-value for interaction of two factors based on ANOVA.

**Table 6:** Reproductive performance of multiparous queens (≥5 litters) in relation to the number of kittens per litter, lifetime number of kittens per queen, interval between litters, and the ratio of females to males per litter (average ± SD).

Breed	Number of Queens	Number of Kittens in Litter	Total Number of Kittens from Queen	Time Between Litters	Ratio of Females in a Litter (%)	Ratio of Males in a Litter (%)
BSH	27	^b^ 4.4 ± 1.8	^a^ 25.9 ± 9.0	^b^ 334.4 ± 133.6	^a^ 49.8 ± 27.3	^a^ 50.2 ± 27.3
MCO	34	^c^ 5.2 ± 2.2	^b^ 33.6 ± 9.6	^a^ 281.5 ± 132.0	^a^ 46.0 ± 24.4	^a^ 54.0 ± 24.4
DRX	7	^a^ 3.3 ± 1.4	^a^ 21.4 ± 6.9	^a^ 252.3 ± 119.7	^a^ 42.5 ± 32.8	^a^ 57.5 ± 32.8
General		4.7 ± 2.1	29.3 ± 10.1	297.9 ± 134.2	47.0 ± 26.6	53.0 ± 26.6
*p*-values *		<0.001	<0.001	<0.001	0.186	0.186

a, b, c—Values in columns marked with different letters are significantly different at *p* ≤ 0.05. Number of queens—all females that produced at least five litters during the analyzed period; * *p*-value for main effect of the first factor (breed) based on ANOVA.

**Table 7:** Pearson correlation coefficient between the age of the queen, the interval between litters, and the number of kittens per litter.

Breed		Queen’s Age (Days)	Time Between Litters (Days)	Number of Kittens in Litter
BSH	Queen’s age (days)		0.273 *	−0.079
	Time between litters (days)	0.273 *		−0.125 *
	Number of kittens in litter	−0.079	−0.125 *	
MCO	Queen’s age (days)		0.294 *	−0.066
	Time between litters (days)	0.294 *		−0.130 *
	Number of kittens in litter	−0.066	−0.130 *	
DRX	Queen’s age (days)		0.387 *	−0.153
	Time between litters (days)	0.387 *		−0.179
	Number of kittens in litter	−0.153	−0.179	
General	Queen’s age (days)		0.304 *	−0.069
	Time between litters (days)	0.304 *		−0.133 *
	Number of kittens in litter	−0.069	−0.133 *	

* Significant correlations at *p* ≤ 0.05.

## Data Availability

The original contributions presented in this study are included in the article. Further inquiries can be directed to the corresponding author.

## Supplementary Materials

The following supporting information can be downloaded at: https://www.mdpi.com/article/10.3390/life15071128/s1.
